# Disability-Adjusted Life Years for Cancer in 2010–2014: A Regional Approach in Mexico

**DOI:** 10.3390/ijerph15050864

**Published:** 2018-04-26

**Authors:** Efrén Murillo-Zamora, Oliver Mendoza-Cano, Mónica Ríos-Silva, Ramón Alberto Sánchez-Piña, Martha Alicia Higareda-Almaraz, Enrique Higareda-Almaraz, Agustin Lugo-Radillo

**Affiliations:** 1 Unidad de Medicina Familiar No. 19, Instituto Mexicano del Seguro Social, Av. Javier Mina 301, Col. Centro, Colima 28000, Colima, Mexico; efren.murilloza@imss.gob.mx; 2 Facultad de Ingeniería Civil, Universidad de Colima, Km. 9.0 Carretera Colima-Coquimatlán, Coquimatlán 28400, Colima, Mexico; 3 Profesora Investigadora Cátedras CONACyT-Universidad de Colima, Centro Universitario de Investigaciones Biomédicas, Colima 28040, Colima, Mexico; mrios@ucol.mx; 4 Center for Health and the Global Environment, Department of Environmental Health, Harvard TH Chan School of Public Health, 401 Park Drive, P.O. Box 15677, 4th Floor West, Suite 415, Boston, MA 02215, USA; rsanchez@hsph.harvard.edu; 5 Jefatura de Servicios de Prestaciones Médicas, Instituto Mexicano del Seguro Social, Álvaro Obregón 184, Col. Centro, Colima 28000, Colima, Mexico; martha.higareda@imss.gob.mx (M.A.H.-A.); enrique.higaredaa@imss.gob.mx (E.H.-A.); 6 CONACYT-Facultad de Medicina y Cirugía, Universidad Autónoma Benito Juárez de Oaxaca, Ex Hacienda de Aguilera S/N, Carretera a San Felipe del Agua, Oaxaca 68020, Oaxaca, Mexico

**Keywords:** neoplasms, burden of illness, YLL, YLD, DALYs

## Abstract

The disability-adjusted life years (DALYs) were used to estimate the regional (state of Colima, Mexico) cancer burden in 2010–2014. The years of life lost (YLL) were estimated with mortality data and years lived with disability (YLD) using incidence data. The DALYs were calculated as the arithmetic addition of YLL and YLD. Sex and cancer site-specific estimations were made and DALY rates were used to identify the leading causes of disease burden. Data from 2532 deaths were analyzed and, for all malignant tumors combined, 18,712.9 DALYs and 20,243.3 DALYs were estimated in males and females respectively. The overall contribution of YLL in DALY estimates was higher among females (93.7% vs. 87.4%). Age-standardized DALY rates (and 95% confidence intervals, CI) per 100,000 inhabitants were used to rank the leading causes of disease burden and, among males, malignant tumors from the prostate, lower respiratory tract, and colon and rectum accounted the highest rates (45.7, 95% CI 32.7–59.3; 37.6, 95% CI 25.7–49.9; and 25.9, 95% CI 16.0–36.1 DALYs). Breast, cervix uteri, and lower respiratory tract cancer showed the highest burden in females (66.0, 95% CI 50.3–82.4; 44.4, 95% CI 31.5–57.7; and 20.9, 95% CI 12.0–30.0 DALYs). The present study provides an indication of the burden of cancer at the regional level, underscoring the need to expand cancer prevention, screening, and awareness programs, as well as to improve early diagnosis and medical treatment.

## 1. Introduction

Cancer is a leading cause of death worldwide. Due to growing aging population and high prevalence of exposure to known risks factors [1], an increase in cancer burden has been observed and it is expected to almost double by 2030 despite the advances in timely diagnosis and medical treatment [2].

The methodology of the Global Burden of Disease study (GBD) provides a comprehensive assessment of major diseases and injuries. The disability-adjusted life years (DALYs) measure the premature mortality (years of life lost, YLL) and spent time in states of reduced health (years lived with disability, YLD) [3]. An age-weighting function is used in DALY computation and values life years differently depending on the age of illness onset [4]. They are a useful analytical tool in cost-effectiveness analysis [5] and are commonly used for comparison purposes [6].

Regional patterns of cancer incidence and mortality rates have been described [7] and, from an economic and health-policy perspective, the regional estimation of cancer burden represents a helpful analytical tool to identify and prioritize decisions related to the implementation and evaluation of preventive strategies [8]. Located in western Mexico, the state of Colima (650,000 inhabitants) [9] has epidemiological information systems that make the computing of DALYs attributable to malignant tumors viable.

This study aimed to estimate the cancer burden in the state of Colima from 2010–2014 using the DALYs as a health measure.

## 2. Materials and Methods

DALYs for cancer were estimated following the methods described by the GBD study [10] as the arithmetic addition of YLL and YLD. First, data from cancer-related mortality was collected from the Statistical and Epidemiological Death Registration (SEDR) from the National System for Epidemiological Surveillance. The SEDR integrates data from issued death certificates in the state of Colima and its function is regulated by specific governmental normative lineaments [11]. The underlying causes of death (International Classification of Diseases 10th revision, ICD-10) included in this analysis were malignant tumors from: mouth and oropharynx (C00–C14); esophagus (C15); stomach (C16); colon and rectum (C18–C21); liver (C22); pancreas (C25); larynx (C32); trachea, bronchus and lung (C33–C34); melanoma and other skin cancers (C43–C44); breast (C50); cervix uteri (C53); corpus uteri (C54–C55); ovary (C56); prostate (C61); bladder (C67); central nervous system (C70–C72); lymphomas and multiple myeloma (C81–C90, C96); leukemia (C91–C95) and other malignant tumors (C00–C97, except for those previously cited). Age-standardized mortality rates (ASMR) per 100,000 inhabitants were estimated using the World Standard Population (2000–2025) [12].

Second, the YLL were computed by multiplying the number of cancer deaths in the study period by the number of expected remaining life at the respective age interval according to the Tables of Life 2013 (Global Health Observatory) from Mexico [13]. The next parameters were fixed: discount rate (*r*) = 0.03, age-weighting (*β*) = 0.04, adjustment constant for age weights (*C*) = 0.1658, and age-weighting modulation (*K*) = 0 [14]. The total population, by sex and age group, was obtained from the National Census of Population and Housing 2010 [9].

Third, the sex-stratified cancer incidence data of Mexico were obtained from the GLOBOCAN project 2012 [15]. The incidence of each malignant tumor was multiplied by the average duration (years) of the disease and the corresponding disability weight to compute the YLD. The disease durations for countries categorized as having ‘established market economics’ were used [10] since this data is not available for Mexico. Two disability weights were employed in DALYs computation: 0.484 (limitation in ≥2 areas; malignant tumors from mouth and oropharynx; esophagus; liver; pancreas; larynx; lower respiratory tract (trachea, bronchus and lung); ovary; bladder and central nervous system) and 0.294 (limitation in 1 area; malignant tumors from stomach; colon and rectum; melanoma and other skin cancers; breast; cervix/corpus uteri; prostate; lymphomas and multiple myeloma; leukemia and other). The assessed functional areas include recreation, education, procreation, and occupation [16].

Finally, the cancer-site DALYs were aggregated to obtain the total estimation by sex and age-standardized DALYs rates per 100,000 inhabitants were used to rank the leading causes of burden of disease; 95% confidence intervals (CI) were computed. Spreadsheets (Microsoft^®^ Excel^®^) from the GBD study [17] were used to compute the parameters of interest and the summary statistics were estimated using Stata^®^ MP 13.0 (StataCorp LP, College Station, TX, USA).

### Ethical Considerations

This study was approved by the Ethics of Health Research Committee. Data regarding the identification of individuals included in the study sample were omitted in order to preserve their anonymity.

## 3. Results

In the study period, a total 20,418 deaths from all causes were registered and data from 2532 cancer-attributable deaths were analyzed (males, 51.8%). The sex-stratified cancer mortality is shown in Table 1. The overall ASMRs were 69.0 and 61.8 deaths per 100,000 inhabitants in males and females respectively. The malignant tumors from the prostate (11.1 deaths per 100,000 inhabitants) and from the breast (11.4 deaths per 100,000 inhabitants) were the most frequent underlying causes of death in males and females as corresponding. Cancer of the lower respiratory tract (trachea, bronchus, and lung) was also a frequent cause of mortality, mainly among male individuals (10.1 vs. 4.1 deaths per 100,000 inhabitants).

Table 2 shows the YLL and YLD by sex and cancer site. For all malignant neoplasms combined, the YLL were higher among females than in males (18,971.6 vs. 16,345.9). The computed YLD were lower among female individuals (1271.7 vs. 2367.0).

Among males, five malignant tumors (from prostate, lower respiratory tract (trachea, bronchus and lungs), colorectal, leukemia, and liver) were responsible of nearly a half (48.4%) of total YLL computed. Breast, cervix uteri, and ovarian cancer contributed to 39.0% of premature life lost among females.

The cancer-attributable DALYs were 18,712.9 and 20,243.3 in males and females as corresponding (Table 3). The overall mean DALYs from all cancer sites was 7791.2 per year. The highest age-standardized DALY rates per 100,000 inhabitants among males were observed in prostate (45.7, 95% CI 32.7–59.3), lower respiratory tract (37.6, 95% CI 25.7–49.9), and colon and rectum (25.9, 95% CI 16.0–36.1) cancer. Malignant tumors from the breast (66.0, 95% CI 50.3–82.4), cervix uteri (44.4, 95% CI 31.5–57.7), and lower respiratory tract (20.9, 95% CI 12.0–30.0) were the leading causes of cancer burden among female participants.

## 4. Discussion

The regional cancer burden from 2010 to 2014 was estimated in this study using DALYs as a health measure, which combines incidence and mortality data. To our best knowledge, this is the first study estimating the disease burden at state-level. Our findings provide quantitative evidence that may be useful for implementation, prioritization, and evaluation of specific health policies focused on the prevention and early diagnosis of malignant tumors.

Cancer is a leading cause of disease burden in Mexico mainly due to a high premature death; the observed contribution of YLL to the overall DALYs estimates in our study was high (87.4% and 93.7% in males and females, respectively). A high proportion of YLL in DALYs for cancer has been previously described in other populations [18,19,20]. Higher cancer-related burden has been in urban areas when compared with rural locations [21]; information regarding place of residence of enrolled individuals was not collected in our study.

We observed that breast and prostate cancer had the highest disease burden (3937.2 and 2730.4 DALYs) among females and males, respectively. A published research ranking the cancer burden among users from the Mexican Institute of Social Security (from Spanish *Instituto Mexicano del Seguro Social*) had similar findings [22]. The YLL proportion in that study was lower than ours (breast, 56.5% vs. 94.1%; prostate, 45.5% vs. 80.9%) maybe due to the existence of institutional protocols (e.g., OncoIMSS) [22] implemented to provide a timely diagnosis and treatment and also to improve the prognosis of cancer patients.

Malignant tumors from the prostate are the leading cause of cancer death among adult males [23] and the mortality rate from the state de Colima, where this study took place, is one of the highest in the country [24]. High occurrence of late stages (Gleason score ≥ 7) at the time of diagnosis have been documented, in a population from northern Mexico [25], and may be determining in the observed mortality. In addition, Mexico lacks a population-based screening program regarding this malignant tumor [26]. Interestingly, economic analyses have shown that early detection of prostate tumors among individuals aged 70 years and older is not cost-effective [27].

Breast cancer is the leading cause of cancer mortality among women of reproductive age and an increase in its incidence has been documented in Mexico [28]. The screening program is based on breast self-examination and mammography among women aged 40–69 years old, however, the screening coverage is low (40–49 years old, 11.7%; 50–59, 22.8%) [28] and more than 80% of breast neoplasms are diagnosed in late clinical stages [29]. A significant role of perception from organizational and structural factors has been observed in both breast and prostate cancer screening use [25,30].

Plausible strategies to decrease the breast cancer burden include the reduction in exposure to environmental risk factors (i.e., alcohol use) [31], promoting healthy life habits [32], the implementation of a well-organized screening program, control of quality in mammography screening, and standardization of medical management protocols [33].

Considering both genders, the lung cancer mortality rate among Mexican adults is the highest [34]. In our study, the computed DALYs were higher among males (2244.4 vs. 1246.7) and this scenario is consistent with the higher prevalence of smoking habit among them [35]. Smoking is a major risk factor for malignant tumors of respiratory tract [36] and smoke-free legislation has been promoted, and a decrease in the exposure to secondhand tobacco smoke has been documented [37]. The former law in Mexico took effect in April 2008 and prohibits smoking in enclosed public spaces (i.e., restaurants, bars, commercial establishments, and public vehicles) [38].

Overweight and obesity are associated with increased risk of malignant tumors [39] and, among Mexican individuals aged 20 years and older, are associated with high prevalence of exposure to excess body weight (overweight, 32.4%; obesity, 38.8%) [40]. In the state of Colima, where this study took place, the cancer-related premature death attributable to overweight and obesity has been assessed [41]. Targeting overweight and obesity may improve cancer prevention and outcomes after diagnosis, also the burden reduction may be potentially observed [42].

The discount rate (*r* = 0.03) and standard age weighting (*β* = 0.04, *C* = 0.1658) were used for DALYs computation in our study. The discount rate reflects the social value of year lived in a state of health and, from a health-economics perspective; age weighting enables to give more value to a year of live in young adulthood than a year in the extremes of live [10]. There is not a general consensus regarding the social weighting and lower DALYs are computed when age weighting and discount rate are employed [14].

In addition, the usefulness of cancer site-specific causes of death reported on death certificates has been evidenced [43]. However, and among patients with multiple cancer sites, attributing the underlying cause of death may be challenging and impacts on the quality of registered data [43].

There are some alternatives to DALYs approach in economic evaluation and they include the healthy year equivalent (HYE) [44], willingness-to-pay (WTP) [45], and the quality-adjusted life year (QALY) [46]. The HYE reflects the utility function of an individual over their lifetime and health states and is a measure of life quality [44]. However, the HYE have been criticized for the difficulty of implementation in practice and its estimation is considered unworkable [47]. The WTP approach uses a cost-benefit framework to obtain, in monetary terms, how much the individuals would be willing to pay to obtain or avoid specific health effects. The ability to pay is closely associated with the WTP evaluation and this approach will disadvantage people with lower incomes [48]. The QALY incorporates the impact on both the quantity and quality of life from a specific health-related event and are widely used in health economics evaluation [49]. However, they do not incorporate equity weights, which may potentially limit the use of QALY for public health interventions [50].

The limitations of our study must be cited. First, only deaths that occurred in the state of Colima were analyzed and these results may not be reproduced in other territories from Mexico, since regional patterns in cancer-related morbidity and mortality have been described [51]. However, the demographic characteristics of the population of Colima are similar to those observed at national level. Second, life expectancy estimates for Mexico were employed in YLL computing instead of standard life expectancy. Mexico is currently going through a deep epidemiological transition [39,52] and the use of specific estimates may result more accurate according to the aim of this study, however this fact may limit the comparability of our findings. The standard life expectancy is approximately 7% lower than the life expectancy of Mexico, and lower YLL and DALYs rates would have been obtained by using global estimates. Third, reliability of cancer incidence and cancer-related mortality are major limitations since Mexico lacks a population-based cancer registry [52]. Incidence data employed on the computing of cancer burden was obtained from the GLOBOCAN project and it was estimated from national mortality data and modelled survival [15]. On the other hand, the local system of death registration is considered one of the best worldwide in terms of quality and integrity of data [42]. The proportion of cancer-attributable deaths in our study (12.4%) was similar to the observed proportion in a national analysis (12.8%) [52]. Fourth, since no year-stratified analyses were performed, the 2010 total population of the state of Colima was employed (according to government data) and no projections were considered. However, and in accordance with official projections, the local average annual population growth (2010–2014) was <2% [53] and it minimizes the overall effect on the estimates. Lower morbidity and mortality rates would have been observed by using denominators from 2014.

## 5. Conclusions

Cancer is a leading cause of disease burden, and regional estimates were provided in this study from a cost-effectiveness perspective using DALYs as a health measure. There is an urgent need to implement effective cancer prevention programs, including screening and awareness. Ensuring access to early diagnosis and treatment is also a need.

## Figures and Tables

**Table 1:** Sex-stratified cancer mortality in the study population, Colima, Mexico 2010–2014.

	Cancer Site	ICD-10	Males		Females	
			Deaths (n)	ASMR ^a^	Deaths (n)	ASMR ^a^
1	Mouth and oropharynx	C00–C14	36	1.9	15	0.7
2	Esophagus	C15	20	1.1	6	0.2
3	Stomach	C16	63	3.5	61	2.7
4	Colon and rectum	C18–C21	91	5.1	66	3.4
5	Liver	C22	93	4.9	84	4.2
6	Pancreas	C25	66	3.6	49	2.3
7	Larynx	C32	48	2.6	3	0.2
8	Lower respiratory tract ^b^	C33–C34	189	10.1	87	4.1
9	Melanoma and other skin cancers	C43–C44	29	1.4	20	0.8
10	Breast	C50	3	0.2	215	11.4
11	Cervix uteri	C53	-	-	144	7.6
12	Corpus uteri	C54–C55	-	-	23	1.2
13	Ovary	C56	-	-	69	3.7
14	Prostate	C61	250	11.1	-	-
15	Bladder	C67	19	0.9	6	0.2
16	Central nervous system	C70–C72	56	3.2	46	2.5
17	Lymphomas and multiple myeloma	C81–C90, C96	64	3.6	37	1.8
18	Leukemia	C91–C95	64	3.8	46	2.7
19	Other malignant tumors	-	221	12.3	243	12.2
	All sites	C00–C97	1312	69.0	1220	61.8

Abbreviations: ICD-10, International Classification of Diseases 10th Revision; ASMR, age-standardized mortality rate. ^a^ Per 100,000 inhabitants; estimated using the World Standard Population (2000–2025). ^b^ Trachea, bronchus, and lung.

**Table 2:** Years of life lost, years lived with disability, and unadjusted rates per 1000 inhabitants, Colima, Mexico 2010–2014.

	Cancer Site	ICD-10	Males				Females			
			YLL (n/Rate)		YLD (n/Rate)		YLL (n/Rate)		YLD (n/Rate)	
1	Mouth and oropharynx	C00–C14	462.8	1.4	87.0	0.3	219.3	0.7	49.6	0.2
2	Esophagus	C15	281.6	0.9	21.2	0.1	73.8	0.2	10.3	0.03
3	Stomach	C16	801.0	2.5	200.4	0.6	775.8	2.4	129.5	0.4
4	Colon and rectum	C18–C21	1254.8	3.9	292.4	0.9	957.3	2.9	220.3	0.7
5	Liver	C22	1134.3	3.5	87.5	0.3	1144.7	3.5	40.9	0.1
6	Pancreas	C25	871.2	2.7	62.3	0.2	633.2	1.9	43.0	0.1
7	Larynx	C32	599.0	1.9	74.0	0.2	51.0	0.2	9.0	0.03
8	Lower respiratory tract ^b^	C33–C34	2141.0	6.6	103.4	0.3	1179.1	3.6	67.6	0.2
9	Melanoma and other skin cancers	C43–C44	311.1	1.0	14.3	0.04	218.9	0.7	10.8	0.03
10	Breast	C50	43.3	0.1	2.1	0.01	3706.0	11.3	231.2	0.7
11	Cervix uteri	C53	-		-		2508.2	7.7	139.0	0.4
12	Corpus uteri	C54–C55	-		-		365.7	1.1	27.6	0.1
13	Ovary	C56	-		-		1190.5	3.6	31.2	0.1
14	Prostate	C61	2208.9	6.8	521.5	1.6	-		-	
15	Bladder	C67	198.9	0.6	34.5	0.1	73.7	0.2	14.8	0.05
16	Central nervous system	C70–C72	841.2	2.6	73.1	0.2	849.2	2.6	61.6	0.2
17	Lymphomas and multiple myeloma	C81–C90, C96	932.4	2.9	97.6	0.3	532.7	1.6	37.4	0.1
18	Leukemia	C91–C95	1179.8	3.7	149.7	0.5	879.2	2.7	87.8	0.3
19	Other malignant tumors	-	3084.6	9.6	546.0	1.7	3613.3	11.0	60.1	0.2
	All sites	C00–C97	16,345.9	50.6	2367.0	7.3	18,971.6	57.9	1271.7	3.9

Abbreviations: ICD-10, International Classification of Diseases 10th Revision; YLL, years of life lost; YLD, years lived with disability. ^b^ Trachea, bronchus, and lung.

**Table 3:** Sex-stratified leading cancer causes of disability-adjusted life years, Colima, Mexico 2010–2014.

Cancer Site		ICD-10	%YLL in DALYs	DALYs			
				n (%)		Rate (95% CI) ^a^	
**Males**							
1	Prostate	C61	80.9	2730.4	(14.6)	45.7	(32.7–59.3)
2	Lower respiratory tract ^b^	C33–C34	95.4	2244.4	(12.0)	37.6	(25.7–49.9)
3	Colon and rectum	C18–C21	81.1	1547.2	(8.3)	25.9	(16.0–36.1)
4	Leukemia	C91–C95	88.7	1329.5	(7.1)	22.3	(13.1–31.7)
5	Liver	C22	92.8	1221.8	(6.5)	20.5	(11.7–29.5)
6	Lymphomas and multiple myeloma	C81–C90, C96	90.5	1030.0	(5.5)	17.3	(9.2–25.5)
7	Stomach	C16	80.0	1001.4	(5.4)	16.8	(8.8–24.9)
8	Pancreas	C25	93.3	933.5	(5.0)	15.6	(7.9–23.5)
9	Central nervous system	C70–C72	92.0	914.3	(4.9)	15.3	(7.7–23.1)
10	Larynx	C32	89.0	673	(3.6)	11.3	(4.7–18.0)
	Other sites	-	86.1	5087.4	(27.2)	85.2	(67.5–103.9)
	All sites	C00–C97	87.4	18,712.9		313.5	(280.4–350.2)
**Females**							
1	Breast	C50	94.1	3937.2	(19.4)	66.0	(50.3–82.4)
2	Cervix uteri	C53	94.7	2647.2	(13.1)	44.4	(31.5–57.7)
3	Lower respiratory tract ^b^	C33–C34	94.6	1246.7	(6.2)	20.9	(12.0–30.0)
4	Ovary	C56	97.4	1221.7	(6.0)	20.5	(11.7–29.5)
5	Liver	C22	96.6	1185.6	(5.9)	19.9	(11.2–28.8)
6	Colon and rectum	C18–C21	81.3	1177.6	(5.8)	19.7	(11.1–28.6)
7	Leukaemia	C91–C95	90.9	967	(4.8)	16.2	(8.4–24.2)
8	Central nervous system	C70–C72	93.2	910.8	(4.5)	15.3	(7.6–23.0)
9	Stomach	C16	85.7	905.3	(4.5)	15.2	(7.6–22.9)
10	Pancreas	C25	93.6	676.2	(3.3)	11.3	(4.8–18.0)
	Other sites	-	95.9	5368.0	(26.5)	89.9	(71.8–109.2)
	All sites	C00–C97	93.7	20,243.3		339.2	(304.8–377.4)

Abbreviations: ICD-10, International Classification of Diseases 10th Revision; YLL, years of life lost; DALYs, disability-adjusted life years; CI, confidence interval. ^a^ Age-standardized DALYs rate per 100,000 inhabitants; estimated using the World Standard Population (2000–2025). ^b^ Trachea, bronchus, and lung.

## Abbreviations

DALYs	disability-adjusted life years
YLL	years of life lost
YLD	years lived with disability
GBD	Global Burden of Disease study
SEDR	Statistical and Epidemiological Death Registration system from Mexico
ASMR	age-standardized mortality rates
HYE	healthy year equivalent
WTP	willingness-to-pay
QALY	quality-adjusted life year
